# Pitt-Hopkins syndrome: report of a case with a TCF4 gene mutation

**DOI:** 10.1186/1824-7288-36-12

**Published:** 2010-02-02

**Authors:** Grazia Taddeucci, Alice Bonuccelli, Ilaria Mantellassi, Alessandro Orsini, Enrico Tarantino

**Affiliations:** 1 Department of Procreation Medicine and Developmental Age, Section of Paediatric Neurology, University of Pisa, Italy; 2 Section of Clinical Genetics, AOUP, Pisa, Italy

## Abstract

**Aims:**

We will discuss the clinical and genetic diagnosis of a child with severe psychomotor delay, who at 3 years of age presented with paroxysms of hyperpnea-apnea and seizures unrelated to breathing anomalies.

**Methods:**

The child underwent genetic (karyotype, FISH telomeres) and neuroradiological (cranial CT and MRI) tests, which proved to be normal. He came under our clinical observation at 3 years and 5 months of age. Due to severe psychomotor delay and facial dysmorphisms we completed the genetic investigations based on his clinical feature and analysis of the available literature.

**Results:**

The presence of severe mental retardation associated with anomalous breathing pattern may suggest the Joubert and Rett syndrome, however these were excluded on the basis of clinical and genetic examination. Angelman syndrome, suspected for facial dysmorphisms and absent language, was also excluded because of the presence of a normal pattern of methylation at SNRPN locus. Another possible diagnosis was the Pitt-Hopkins Syndrome (PHS), characterized by severe mental retardation, breathing anomalies (paroxisms of hyperpnea-apnea), dysmorphisms and sometimes epilepsy. Haploinsufficiency of TCF4 gene located at 18q21.2 region has been recently identified as causative of this syndrome. In our patient the research of TCF4 mutation by the Institute of Human Genetics, University Hospital Erlangen (Germany), showed a de novo mutation.

**Conclusions:**

The diagnosis of Pitt-Hopkins syndrome, an underdiagnosed cause of mental retardation, was based on clinical and genetic findings. Searching for TCF4 mutations is highly recommended when others overlapping syndromes was excluded. At our knowledge our patient is the first italian case of PHS diagnosed at molecular level.

## Background

Pitt-Hopkins Syndrome (PHS) is a rare cause of severe mental retardation. First detected in 1978 in two patients [1], at present there are 52 cases reported [1-13]. Typical facial dysmorphisms include a broad and beaked nose, flared nostrils, a wide mouth with a "Cupid's bow" shaped upper lip, cupped ears, broad helices, a broad palate and clubbed fingertips (due to chronic hypossiemia). The patients have severe psychomotor delay and language impairment, postnatal growth retardation and microcephaly. This syndrome is characterized by a particular breathing pattern which appears in mid-childhood and manifests as paroxysms of hypernea followed by apnea and occasionally cyanosis. This pattern occurs during wakefulness and is not associated with epileptic changes, but is increased by emotions or fatigue. Epileptic seizures occur frequently and can be severe; electroencephalographic pattern is often characterized by frontal slow and sharp wave discharges. Cerebral MRI may shows brain anomalies such as bulging caudate nuclei, hypoplastic corpus callosum, small hippocampus and cerebellar vermis hypoplasia. Another frequent symptom is constipation [6-13]. In 2007 Pitt-Hopkins Syndrome's gene was identified by three unrelated groups at 18q21.2 by comparative genomic hybridization (CGH) array. In particular the haploinsufficiency of the TCF4 (transcription cell factor 4) gene, due to an autosomal dominant de novo mutation, is considered to be causative [6-8]. We report a 3 1/2 year old child with severe psychomotor delay, absence of language development, breathing anomalies and facial dysmorphisms, suggestive for diagnosis of Pitt-Hopkins Syndrome.

## Methods

The child was thoroughly examined with anamnestic investigation, clinical evaluation, electroencephalographic profiling. The patient, previously tested for karyotype and FISH telomeres, was reassessed for high resolution chromosome analysis and molecular investigations on Rett (MECP2 gene) and Angelman (methylation test at the SNRPN locus) syndromes. Subsequently DNA samples of patient and his parents were submitted to Institute of Human Genetics, University of Erlangen, Germany, to perform mutational analysis of TCF4 gene by direct sequencing [10].

## Results

**Figure 1:**
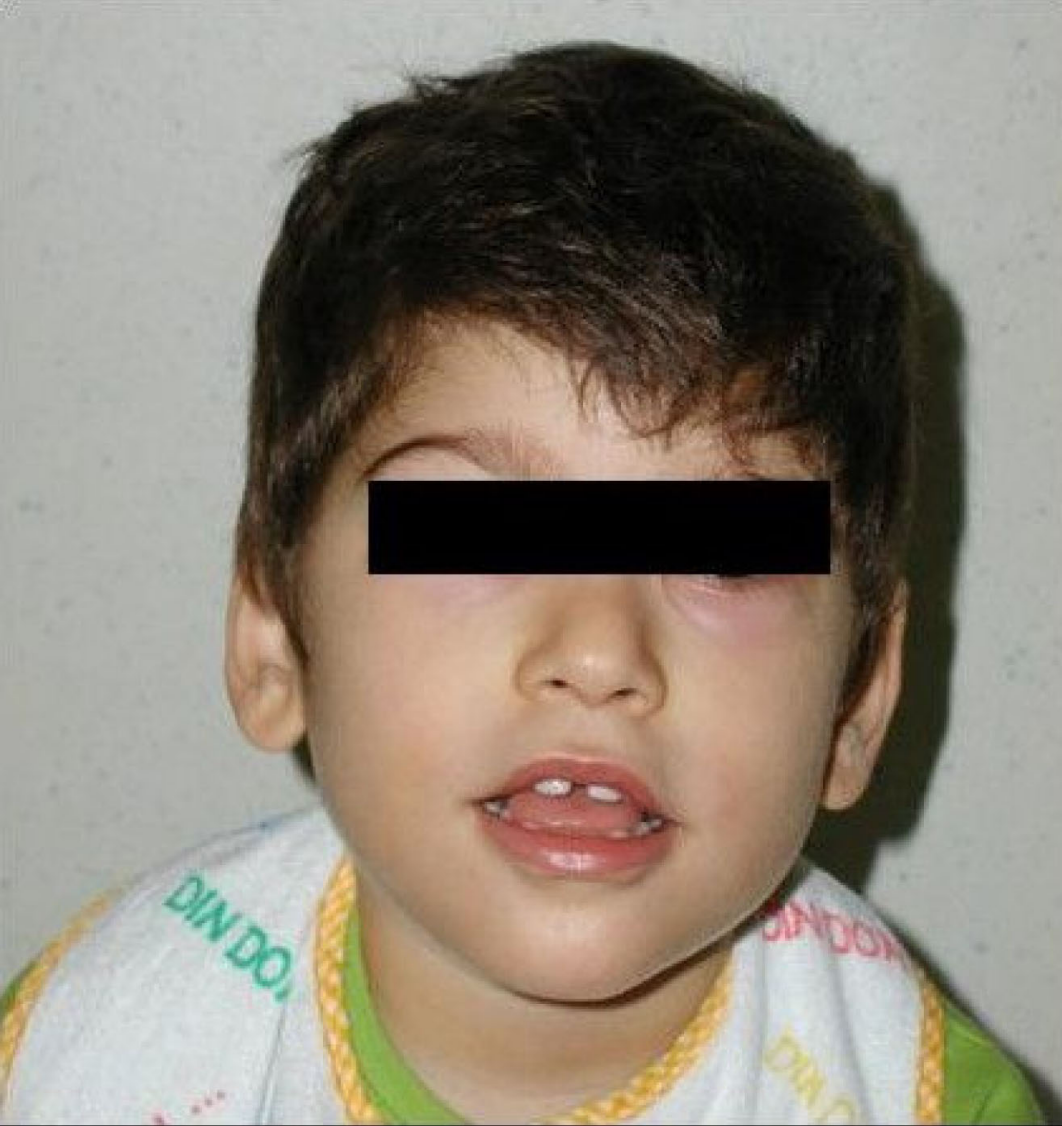
**Note facial dysmorphisms: mycrocephalia, wide mouth with Cupid bow shaped upper lip, narrow biparietal diameter, epicanthus, prognathism**.

**Figure 2:**
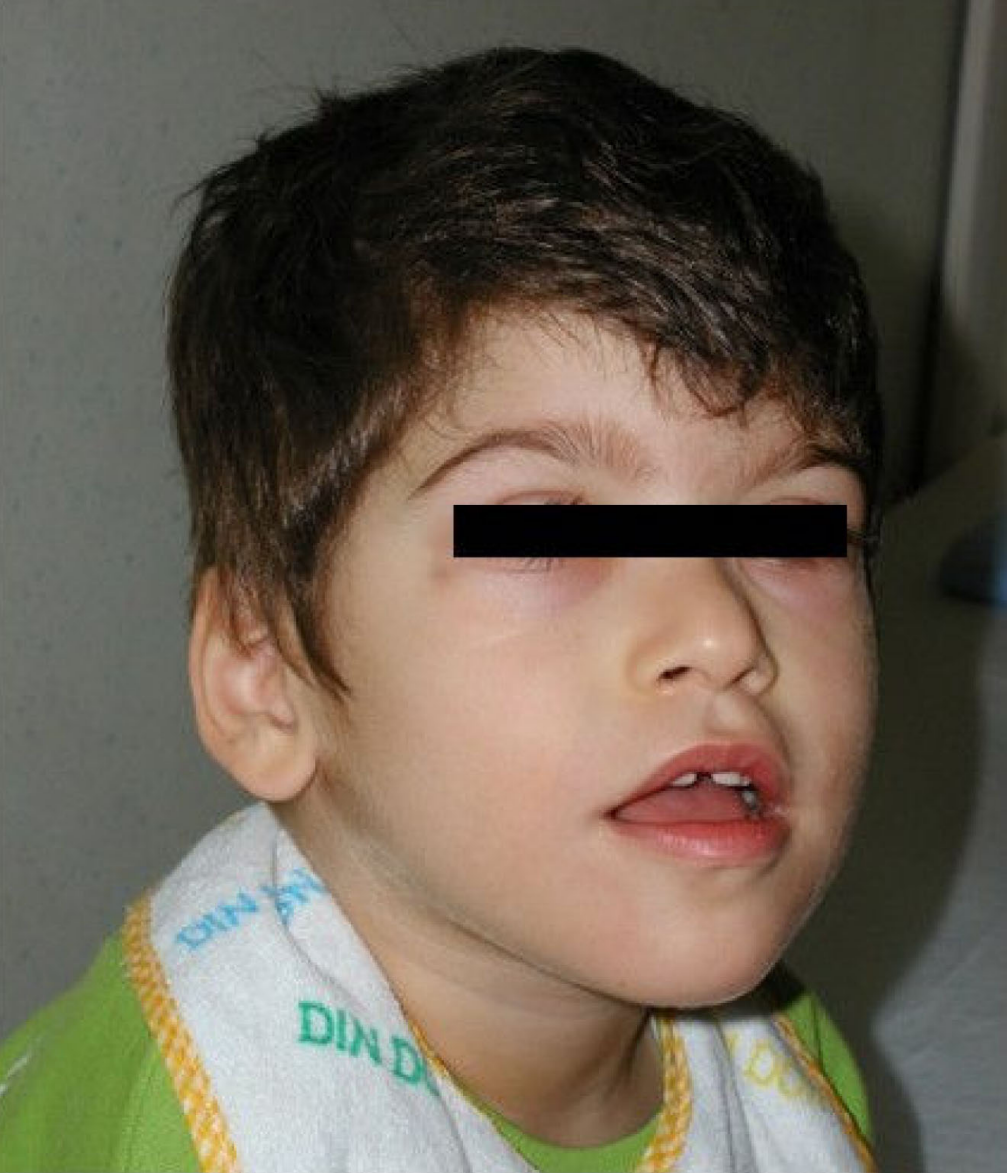
**note facial dysmorphisms: mycrocephalia, wide mouth with Cupid bow shaped upper lip, narrow biparietal diameter, prognathism**.

**Figure 3:**
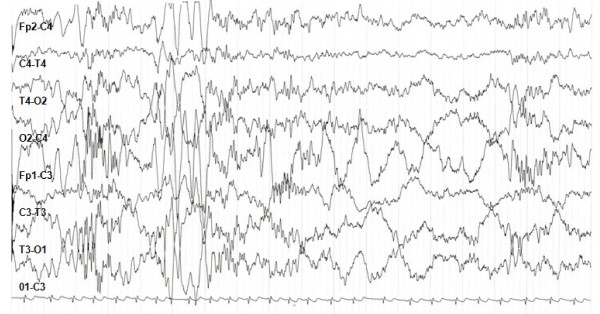
**Sleeping EEG: epileptic anomalies in the frontocentral regions with left prevalence and tendence to the controlateral diffusion**.

The boy, first son of healthy, unrelated parents, was born at full term after a normal pregnancy. Birth weight was 2950 g (<10%), height 48 cm (10%), head circumference 31 cm (< 2 standard deviations). The neonatal course was regular. During the first year of his life, severe psychomotor delay became evident. The ability to sit was acquired after 10 months, walking at 24 months and is still unstable. Language development is absent. The child exhibits stereotypical hand movements, cheerful behavior, and constipation. At 3 years and 2 months, he began to experience daily episodes of tachypnea followed by transitory breathing arrest and cyanosis. Not long after, the child presented with short seizures characterized by loss of axial tone, perioral cyanosis, staring and a fixed smile. Sometimes seizures followed the breathing anomalies. Previously executed metabolic screening and genetic tests (karyotype, FISH telomeres), proved to be normal. The ocular examination, abdominal and cardiac ultrasounds, and rachis radiogram were normal as well. At 2 years and 2 months of age, neuroradiological tests (cranial CT and MRI) showed no abnormality, and an authoritative genetic counseling came to this conclusion: "probable autosomal recessive psychomotor retardation in patient with microcephaly and dysmorphic features". The child was 3 years and 5 months old when he came under our clinical observation. Dysmorphic features were evident: microcephaly (head circumference 47 cm, < 3%), weight 13 kg (<10%), height 94 cm (10%), wide mouth with a "Cupid's bow" shaped upper lip, narrow biparietal diameter, epicanthic fold and prognathism (Figures 1, 2). The sleeping EEG shows anomalies in the frontocentral regions with left prevalence and tendency toward contralateral diffusion (Figure 3). The child underwent anti-epileptic therapy (carbamazepine), resulting in a rapid and dramatic reduction of the seizures. Further genetic tests were carried out: MECP2 analysis and SNRPN methylation pattern produced normal results. Attention then was paid to PHS and patient's DNA was sent to the Institute of Human Genetics in Erlangen (Germany): analysis of the TCF4 gene showed the presence of a de novo heterozygous mutation in the last exon 19: c.1952-1957delCT,p.S661fs, which has not been previously reported (patient eight of the paper of Zweier et al, 2008) [10]. The original stop codon at amino-acid position 672 is altered and the protein associated is elongated by 37 amino-acids.

## Discussion

The presence of severe mental retardation associated with a particular breathing pattern can suggest some genetic syndromes. Joubert Syndrome was excluded because the breathing anomalies begin during neonatal period and later show improvement. Moreover, cerebellar vermis agenesis, ataxy and abnormal eye movements in our patient are absent. Severe mental retardation, breathing anomalies, characteristic hand movements, epilepsy, microcephaly and dysmorphic features all could also suggest Rett Syndrome. This syndrome was excluded because of the different clinical course, facial phenotype and the absence of characteristic hand movements (hand-washing) and MECP2 gene mutations. The presence of epilepsy associated with mental retardation, absent language, occasional laughter, microcephaly, and facial dysmorphisms could also suggest Angelman Syndrome; however this was excluded due to the presence of a normal pattern of methylation at SNRPN locus (region 15q11-q12). Once these syndromes were ruled out, we have considered as a possible diagnostic hypothesis the Pitt-Hopkins Syndrome, for which our patient presents evocatives findings: mental retardation with absent language, typical facial dysmorphic features, breathing pattern which appeared in midchildhood, postnatal growth retardation, microcephaly, epilepsy, electroencephalographic frontal anomalies, manual stereotipies and constipation. Analysis of the TCF4 gene confirmed the diagnosis of Pitt-Hopkins Syndrome. Pitt-Hopkins Syndrome's identification in our case allows to change risk recurrence from likely 25% to a negligible one.

The TCF4 gene, 360 kb in size, encodes for two isoforms of a protein belonging to the class I basic helix-loop-helix (bHLH) protein family, also known as the "E-protein family", which function as transcriptional regulators. This gene is widely expressed in the fetal and adult brain, therefore a TCF4 dysfunction should play a role in the development of mental retardation and epilepsy. TCF4 is also expressed in skeletal muscle, cardiac muscle and lungs. Therefore a TCF4 mutation is consistent with post-natal growth retardation and microcephaly. Autonomic dysfunctions such as hyperventilation and constipation could be ascribed to TCF4 dysregulation, since this gene is involved in the development of the noradrenergic system neurons [6,7]. From the analysis of the literature, until today 219 cases were tested for TCF4 mutations for clinical suspicion of Pitt-Hopkins Syndrome; the two original cases published by Pitt and Hopkins and the case described by Sigh were not tested because not available [7]. In 52/219 (24%) the diagnosis of Pitt-Hopkins Syndrome was molecularly confirmed (table 1). So it is possible that other genes or mutations in regulatory regions of TCF4 could be involved in this syndrome.

**Table 1:** Cases of Pitt-Hopkins syndrome reported in the literature and TCF4 gene mutations.

Authors	PHS phenotype	Molecular analysis	Cases with TCF4 mutation
Pitt D-Hopkins I, 1978	n°2	-	-

Singh HA, 1993	n°1	-	-

Van Balkom ID et al, 1998	n°1	n°1	-

Orrico A et al, 2001	n°2	n°2	-

Peippo MM et al, 2006	n°2	n°2	n°2

Amiel J et al, 2007	n°4	n°4	n°4

Brockschmidt A, 2007	n°1	n°1	n°1

Zweier C et al, 2007	n°24	n°24	n°4

Andrieux J et al, 2008	n°1	n°1	n°1

Zweier, 2008	n°117	n°117	n°16

Kalscheuer, 2008	n°1	n°1	n°1

Giurgea, 2008	n°30	n°30	n°10

De Pontual, 2009	n°36	n°36	n°13

**Tot.**	**222**	**219**	**52**

A significative segnalation is the case of a girl with mental retardation and TCF4 mutation but without the classical picture of Pitt-Hopkins Syndrome; however in this case the genetic impairment is due to balanced translocation and that could not result in a complete haploinsufficiency [11]. This report suggests that TCF4 gene may be involved also in milder clinical phenotype and that further genotypic-phenotypic correlation is necessary in patient with TCF4 mutation.

In conclusion this syndrome should be suspected on the basis of clinical findings even before the occurrence of characteristic breathing patterns and epilepsy [6]. In this regard, the breathing pattern, although highly specific for the syndrome, is reported in 32 out of 52 cases with molecular diagnosis of Pitt-Hopkins Syndrome, about 62%, very similar to the percentage reported by Zweier et al [10]. We agree with general impression that Pitt-Hopkins Syndrome is widely underdiagnosed. TCF4 mutation analysis results highly recommended in patients with severe development delay, absent or very limited speech, breathing anomalies and typical facial gestalt, after the exclusion of the other overlapping, more common syndromes (Angelman, Rett, Joubert).

At our knowledge this is the first italian case of PHS diagnosed at molecular level.

## Consent

Written informed consent was obtained from the patient's parents for publication of this case report and accompanying images. A copy of the written consent is available for review by the Editor-in-Chief of this journal.

## Competing interests

The authors declare that they have no competing interests.

## Authors' contributions

GT has definied the clinical picture of the patient (severe psychomotor delay, breathing anomalies and facial dysmorphisms) and formulated the diagnostic suspicion of the Pitt-Hopkins syndrome.

AB has been involved in the collection of clinical data of the patient and in drafting the manuscript.

IM has been involved in the collection of clinical data of the patient and in drafting the manuscript.

AO has been involved in the collection of clinical data of the patient and in drafting the manuscript.

ET has definied the genetic hypothesis of Pitt-Hopkins syndrome and sent the blood sample of the patient to University Hospital Erlangen, Germany, for the genetic diagnosis.
